# Atherosclerosis is associated with a decrease in cerebral microvascular blood flow and tissue oxygenation

**DOI:** 10.1371/journal.pone.0221547

**Published:** 2019-08-30

**Authors:** Baoqiang Li, Xuecong Lu, Mohammad Moeini, Sava Sakadžić, Eric Thorin, Frederic Lesage

**Affiliations:** 1 Institute of Biomedical Engineering, École Polytechnique de Montréal, Montréal, QC, Canada; 2 Research Center, Montreal Heart Institute, Montréal, QC, Canada; 3 Department of Biomedical Engineering, Amirkabir University of Technology (Tehran Polytechnic), Tehran, Iran; 4 Athinoula A. Martinos Center for Biomedical Imaging, Massachusetts General Hospital, Harvard Medical School, Charlestown, MA, United States of America; 5 Department of Surgery, Faculty of Medicine, University of Montreal, Montréal, QC, Canada; University of Warwick, UNITED KINGDOM

## Abstract

Chronic atherosclerosis may cause cerebral hypoperfusion and inadequate brain oxygenation, contributing to the progression of cognitive decline. In this study, we exploited two-photon phosphorescence lifetime microscopy to measure the absolute partial pressure of oxygen (PO_2_) in cortical tissue in both young and old LDLR^-/-^, hApoB100^+/+^ mice, spontaneously developing atherosclerosis with age. Capillary red-blood-cell (RBC) speed, flux, hematocrit and capillary diameter were also measured by two-photon imaging of FITC-labelled blood plasma. Our results show positive correlations between RBC speed, flux, diameter and capillary-adjacent tissue PO_2_. When compared to the young mice, we observed lower tissue PO_2_, lower RBC speed and flux, and smaller capillary diameter in the old atherosclerotic mice. The old mice also exhibited a higher spatial heterogeneity of tissue PO_2_, and RBC speed and flux, suggesting a less efficient oxygen extraction.

## Introduction

Chronic atherosclerosis has a lifelong impact on brain health [1–9]. It can cause cerebral hypoperfusion and cerebrovascular disorders [10–12], which, in turn, impairs normal cognitive function [12–15]. Reduced supply of oxygen to brain tissue due to the atherosclerosis-related disruption in cerebral blood flow may induce neurodegeneration [16,17], leading to cognitive decline. The cerebral capillary network is the major site of vessel-tissue oxygen exchange [18]. The role of capillaries in oxygen delivery was emphasized in several modeling studies showing that brain tissue oxygenation benefits from not only the increase in absolute capillary blood flow but also from its homogenization [19–21]. Therefore, alterations of brain tissue oxygenation, and capillary structural and functional properties can indicate deviations from normal brain condition.

Little is known, however, about the distribution and change in brain tissue oxygenation, and capillary blood flow with the progression of atherosclerosis. Furthermore, limited experimental data has been published demonstrating how capillary blood flow and flow spatial patterns modulate oxygen extraction. The recent synthesis of an oxygen-sensitive phosphorescence probe PtP-C343, which can be combined with two-photon phosphorescence lifetime microscopy, enabled measurements of the absolute partial pressure of oxygen (PO_2_) in the parenchymal tissue of brain at the micron scale [22–24]. Exploiting this key development, we aim here to experimentally characterize the distribution of brain tissue oxygenation and cortical capillary blood flow, as well as to investigate the relation between capillary blood flow homogenization and brain tissue oxygenation, in a mouse model of atherosclerosis.

Based on two-photon lifetime imaging of the phosphorescence from the probe PtP-C343 [22,23], we measured absolute partial pressure of oxygen (PO_2_) in the cortical tissue of young and old dislipidemic LDLR^-/-^ mice [25–28]. Capillary red blood cell (RBC) speed, flux, hematocrit and diameter were also measured by two-photon imaging of FITC-labelled blood plasma. We first investigated whether there were correlations between the capillary structural/flow properties and adjacent brain tissue oxygenation in young mice with limited pathology. Performing the same measures in an older group, we then studied age-related differences in cortical tissue PO_2_, capillary RBC speed and flux, the degree of capillary blood flow heterogeneity, and capillary diameter.

## Materials and methods

### Experimental setup

**Fig 1:**
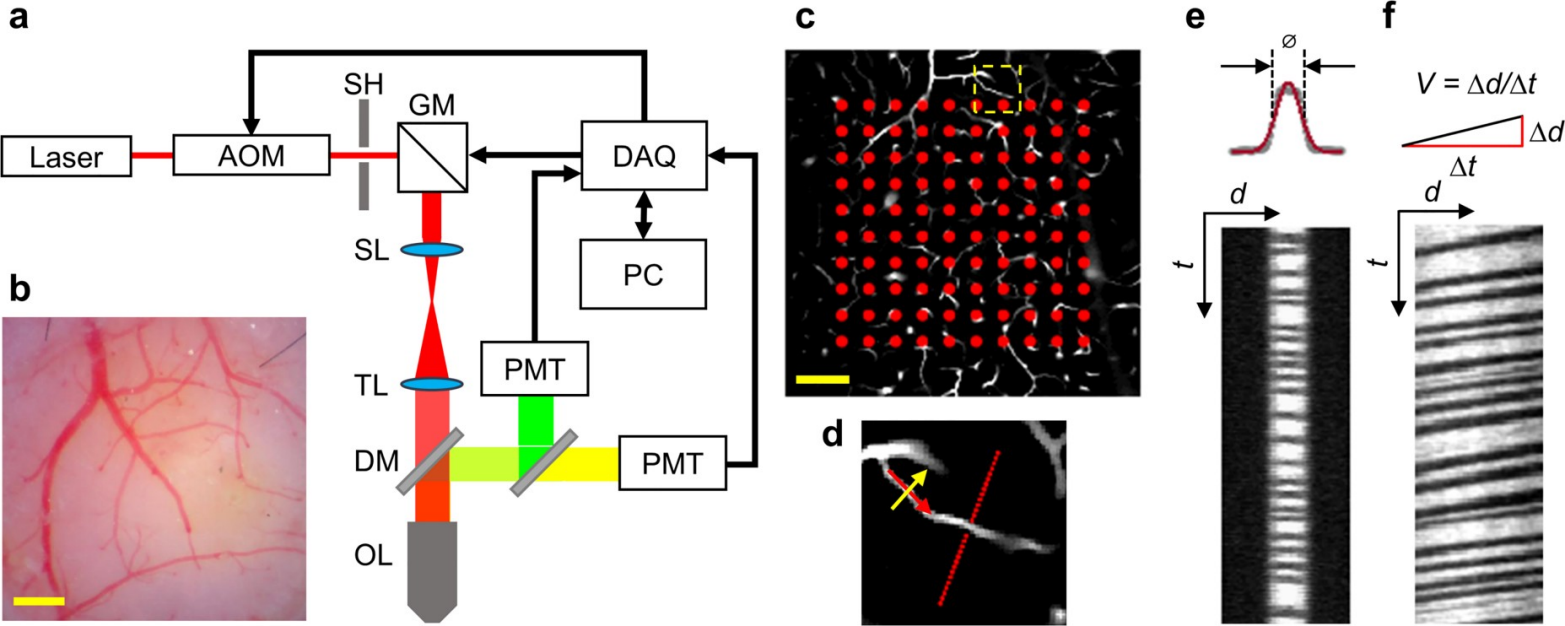
Experimental setup and acquisition protocol. **a.** Schematic diagram of the two-photon microscopy system. The components are abbreviated as: acousto-optic modulator (AOM), shutter (SH), Galvo mirror (GM), scan lens (SL), tube lens (TL), dichroic mirror (DM), objective lens (OL), photomultiplier tube (PMT), data acquisition card (DAQ), and personal computer (PC). **b.** A representative CCD image capturing the mouse brain surface vasculature. Scale bar: 500 μm. **c.** Illustration of the 2D grid of locations (red dots) marked for PO_2_ acquisition performed in the cortical tissue within the capillary bed. The red dashed square encloses a capillary, used as an example of blood flow and tissue PO_2_ gradient acquisition in **d**. Scale bar: 50 μm. **d.** The red dots in the vascular image illustrate the measurement locations for tissue PO_2_ gradient, perpendicular to the capillary axis. In the same capillary, perpendicular and longitudinal line-scans were performed, as illustrated by the yellow and red arrows, respectively. The arrows indicate the line-scan directions. **e** and **f.** Representative perpendicular (**e**) and longitudinal (**f**) line-scan images utilized to extract capillary RBC flow properties and diameter. In **e**, ø, d and t represent diameter, distance and time, respectively. In **f**, the RBC speed V was calculated by the ratio of the distance interval (Δd) to the time interval (Δt).

Experiments were conducted with a home-built two-photon laser scanning microscope (Fig 1A). The system details have been described in previous publications [29,30]. Briefly, we employed a laser (80 MHz, 150-fs pulse width, Mai-Tai, Newport) as a two-photon excitation source. The laser power and gating were controlled by an electro-optic modulator (EOM). The laser beam was raster-scanned by a pair of galvanometric scanners. After the galvo mirrors, the laser beam was magnified by a telescope, consisting of a scan lens and a tube lens, and then imaged on the back-aperture of an objective lens (20×, NA = 1.0, water immersed, XLUMPLFLN 20XW, Olympus). Finally, the beam was focused onto the sample for imaging. The emitted photons were filtered by a dichroic mirror (FF685-DI02-25×36, Semrock) and a band-pass filter (FF02-809/81-25, Semrock), and detected by a photomultiplier tubes (PMTs). Phosphorescence from the PtP-C343 probe was detected by one PMT (H7422, Hamamatsu). Fluorescence from a FITC-labeled blood plasma was detected by another PMT (R3896, Hamamatsu). Acquisition was controlled by a home-developed MATLAB-based software.

### Animal preparation

In this study, n = 6 young (3–4 months, male, 21–28 g) and n = 6 old (15–20 months, male, 35–45 g) transgenic mice from our established colony were used. This transgenic model (wild-type strain: hybrid of 129SvyEv and C57BLy6) has a deletion of the LDL receptor gene and overexpresses human apo-lipoprotein B (LDLR^-/-^; hApoB100^+/+^). Mice lacking the LDL receptor develop atherosclerosis spontaneously without the need of high cholesterol diet [25,26]. The mouse colony has been established for the last 15 years at the Montreal Heart Institute and with these mice consistent levels of atherogenesis have been reported through the years [27,28,31–35]. In the previous studies with this model, it was shown that the atherosclerotic plaque was not significantly developed in the 3-month-old atherosclerotic mice but that plaque area increased significantly in the atherosclerotic mice starting from 6 months [28,33,34]. Learning ability, evaluated with the Morris water maze test, was preserved in the 3-month-old mice, but declined significantly starting from 6-month old, as compared to their age-matched controls [28].

For experiments, mice were first tracheotomised to facilitate animal respiration. Following tracheotomy, a femoral artery cannulation was performed to measure arterial blood gases. Then, an open-skull craniotomy (3 mm in diameter) was done over the primary somatosensory area in the left hemisphere. After the craniotomy, a solution of PtP-C343 (0.6–1.2 μL at 150 μM) was microinjected into brain tissue at 200 μm depth below the brain surface. The cranial window was then filled with agarose diluted in artificial CSF, sealed using a 5-mm diameter microscope coverslip, and the animal was transferred to the two-photon microscope for imaging. Dextran-conjugated FITC (100–200 μL at 50 mg/mL, FD2000S Sigma) was then administered intravenously to visualize micro-vessels enabling localization of the capillary bed for PO_2_ imaging and to measure capillary RBC flow properties. The animals were anesthetized by intraperitoneal administration of urethane (1–1.5 g/kg at 10% W/V) throughout all surgical and experimental procedures. Anesthesia was confirmed by the loss of withdrawal response to toe pinch, and could last for ≥4 hours, long enough for performing both surgery and imaging experiment. During all the surgical procedures and imaging, mice were spontaneously breathing an air/oxygen mixture (air mixed with 10–20% oxygen). The mice were euthanized after experiments using CO_2_ inhalation. All the surgical and experimental procedures were performed in accordance with the Canadian Council on Animal Care recommendations, and were approved by the animal ethics committee of the Montreal Heart Institute research center (Protocol No: 2013–1619).

Prior to imaging, arterial blood gases were measured by a commercial blood gas analyzer to insure physiological levels. In the young group, arterial partial pressure of oxygen (P_a_O_2_) was 102.3±1.4 mmHg; arterial partial pressure of carbon dioxide (P_a_CO_2_) was 37.7±3.2 mmHg; and arterial oxygen saturation (SO_2_) was 97.5±0.3%. In the old group, P_a_O_2_ was 122.0±3.5 mmHg; P_a_CO_2_ was 40.2±3.0 mmHg; and SO_2_ was 98.0±0.6%. Body temperature was maintained at ∼37°C in all mice throughout all experiments. Data are expressed as mean±SEM.

### Data acquisition

#### Tissue PO_2_ imaging

A CCD image of the mouse brain surface vasculature was recorded to guide the selection of a region of interest (ROI) for subsequent two-photon imaging (Fig 1B). PO_2_ measurements were collected typically at two depths, spanning from 150 μm to 250 μm below the brain surface. At each imaging depth, we first performed a raster-scan of FITC intensity, which revealed the location of the capillaries within a 400 × 400 μm^2^ field of view (FOV). Two sets of tissue PO_2_ measurements were collected. First, PO_2_ measurements were performed in a 2D grid geometry (e.g., 10 × 10 measurement locations as denoted by red dots in Fig 1C) in the parenchymal tissue of the cortical capillary bed. Second, PO_2_ was measured in tissue as a function of distance from the capillary wall in the direction perpendicular to the longitudinal axis of the capillary, with sub-micron steps, over a radial distance of 30 μm (Fig 1D).

At each measurement location, the phosphorescent nanoprobe (PtP-C343) in the focal volume was excited with a 25-μs-long laser excitation at 820 nm gated by the EOM, followed by a 275-μs-long collection of the emitted phosphorescence. Typically, at each location, 3,000 such 300-μs-long excitation/decay cycles were repeated to obtain an average phosphorescence decay with sufficient signal-to-noise ratio (SNR) for accurate lifetime calculation. Further technical details have been described in [23,29].

#### Imaging of capillary RBC flow

In the same capillaries for which PO_2_ gradients were measured in the adjacent tissue, RBC speed, flux and hematocrit were measured by performing longitudinal and perpendicular line-scans (Fig 1E and 1F) [36,37] on the FITC-labelled capillaries. The line-scan sampling frequency was 800 Hz, fast enough to accurately measure RBC speed and flux in most cortical capillaries in mice without aliasing effect [38]. The line-scans and tissue PO_2_ gradients were acquired sequentially in time, capillary by capillary. The combined acquisitions for each capillary were completed within ≤10 seconds.

### Data analysis

#### Calculation of PO_2_

The phosphorescence lifetime was calculated by fitting the average 275-μs phosphorescence decay to a single exponential decay function, using a non-linear least square minimization algorithm [22,23,29]. The lifetime was then converted to absolute PO_2_ using a Stern-Volmer type calibration plot obtained in an independent oxygen titration experiment.

#### Capillary PO2 gradient and wall PO_2_

**Fig 2:**
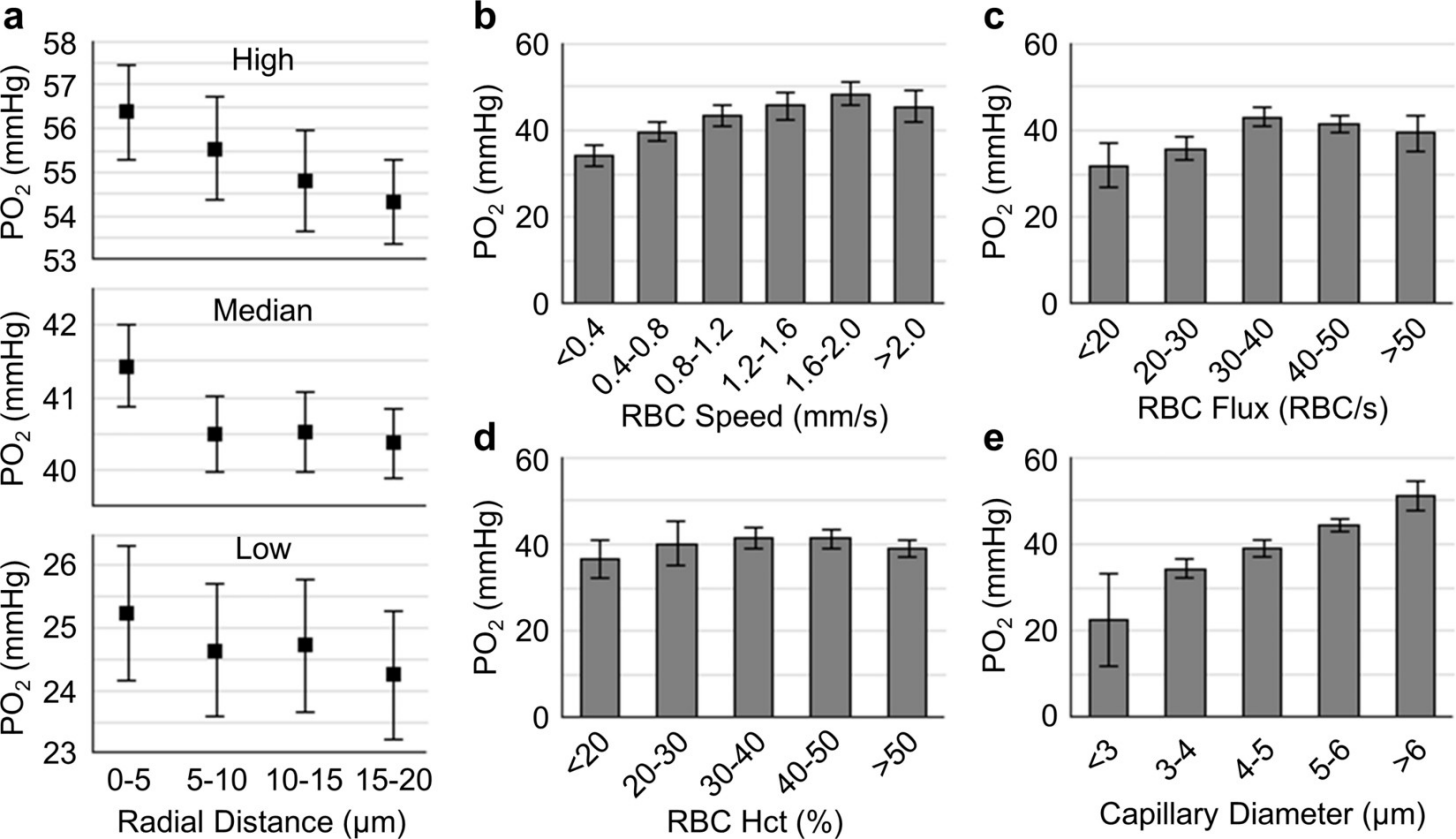
Relations between capillary RBC flow properties, diameters, wall PO_2_, and adjacent tissue PO_2_ gradients. **a.** Average tissue PO_2_ gradients from high, medium and low wall PO_2_ capillary groups, collected across n = 12 mice. **b**-**e.** Relations between RBC speed, flux, hematocrit (Hct), diameter and wall PO_2_. The analysis in **b**-**e** was made with the measurements acquired in ~15 capillaries per mouse across n = 12 mice (including both the young and old mice). Data are expressed as mean±SEM.

To observe the capillary PO_2_ gradient, the PO_2_ values were binned with 5-μm bins (Fig 2A). Capillary wall PO_2_ was calculated based on tissue PO_2_ measured radially in respect to the capillary axis, by averaging the PO_2_ values within the first 10 μm from the capillary wall.

#### Calculation of capillary RBC flow properties and diameter

RBC flux was calculated by counting the number of the dark shadows in the perpendicular (Fig 1E) or longitudinal (Fig 1F) line-scan images during the acquisition time (*t*). The angle of the dark streaks in the longitudinal line-scan image (Fig 1F) was estimated to calculate the RBC speed as Δ*d*/Δ*t*, where Δ*d* and Δ*t* are the distance and time intervals, respectively. RBC hematocrit was computed as the ratio (in %) of the number of pixels in the dark shadows to the total number of pixels in the longitudinal line-scan image. Capillary diameter was calculated as the full width at half maximum, by fitting the intensity profile along the distance axes (*d*) of the perpendicular line-scan image with a Gaussian model (Fig 1E). More details about the line-scan acquisition parameters can be found in [29].

#### Calculation of RBC flow and tissue PO_2_ heterogeneity

For each of the measured parameters, its heterogeneity was quantified as standard deviation (STD) and coefficient of variation (CV) of its data population. Here, CV was calculated as the ratio of STD to mean [39].

#### Statistical analysis

**Fig 3:**
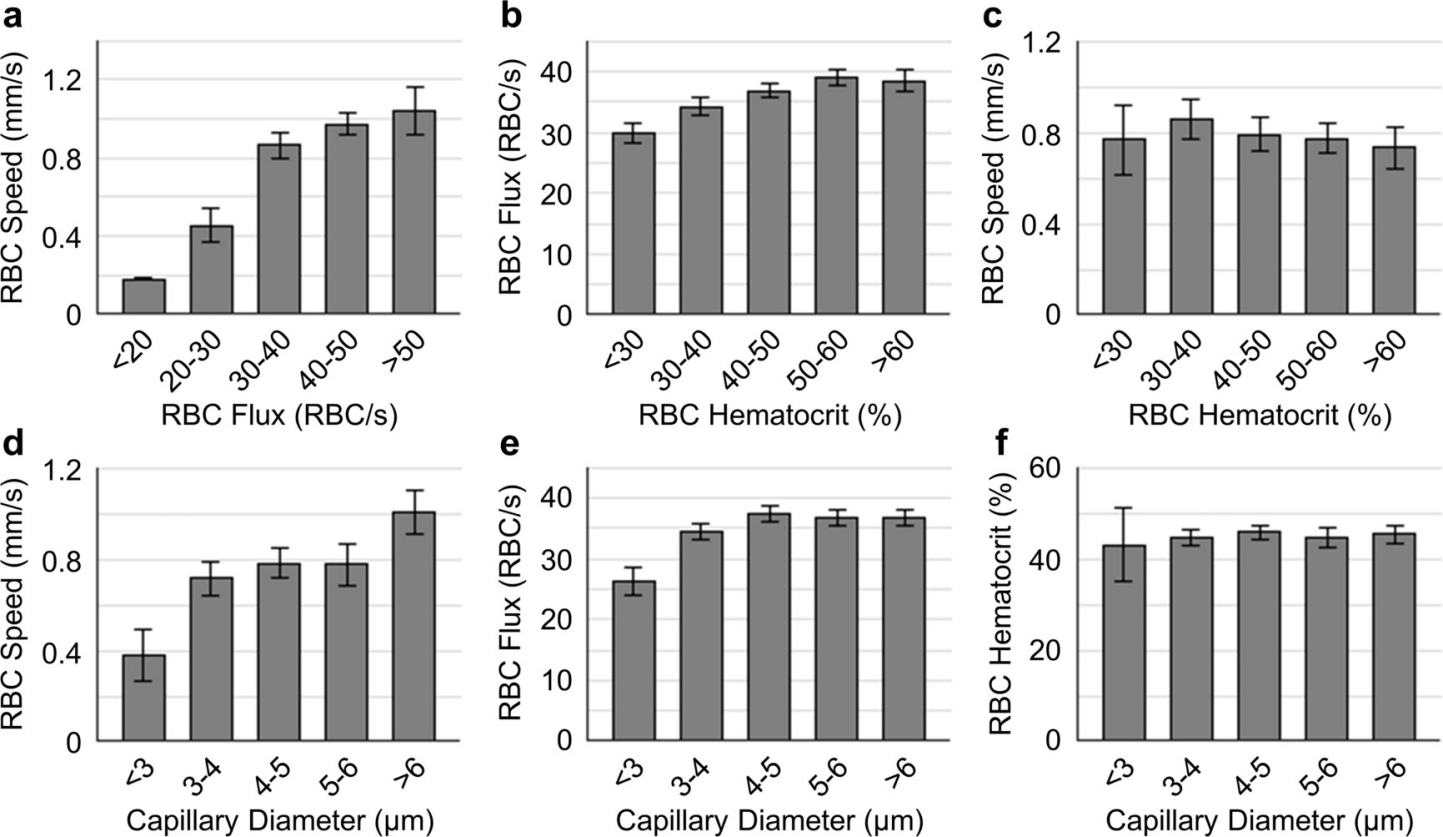
Pairwise relations between capillary RBC flow properties and diameters. **a-f.** Pairwise relations between RBC speed, flux, Hct and diameter. The analysis in **a**-**f** was made with the measurements acquired in ~15 capillaries per mouse, across n = 12 mice (including both the young and old mice). Data are expressed as mean±SEM.

**Fig 4:**
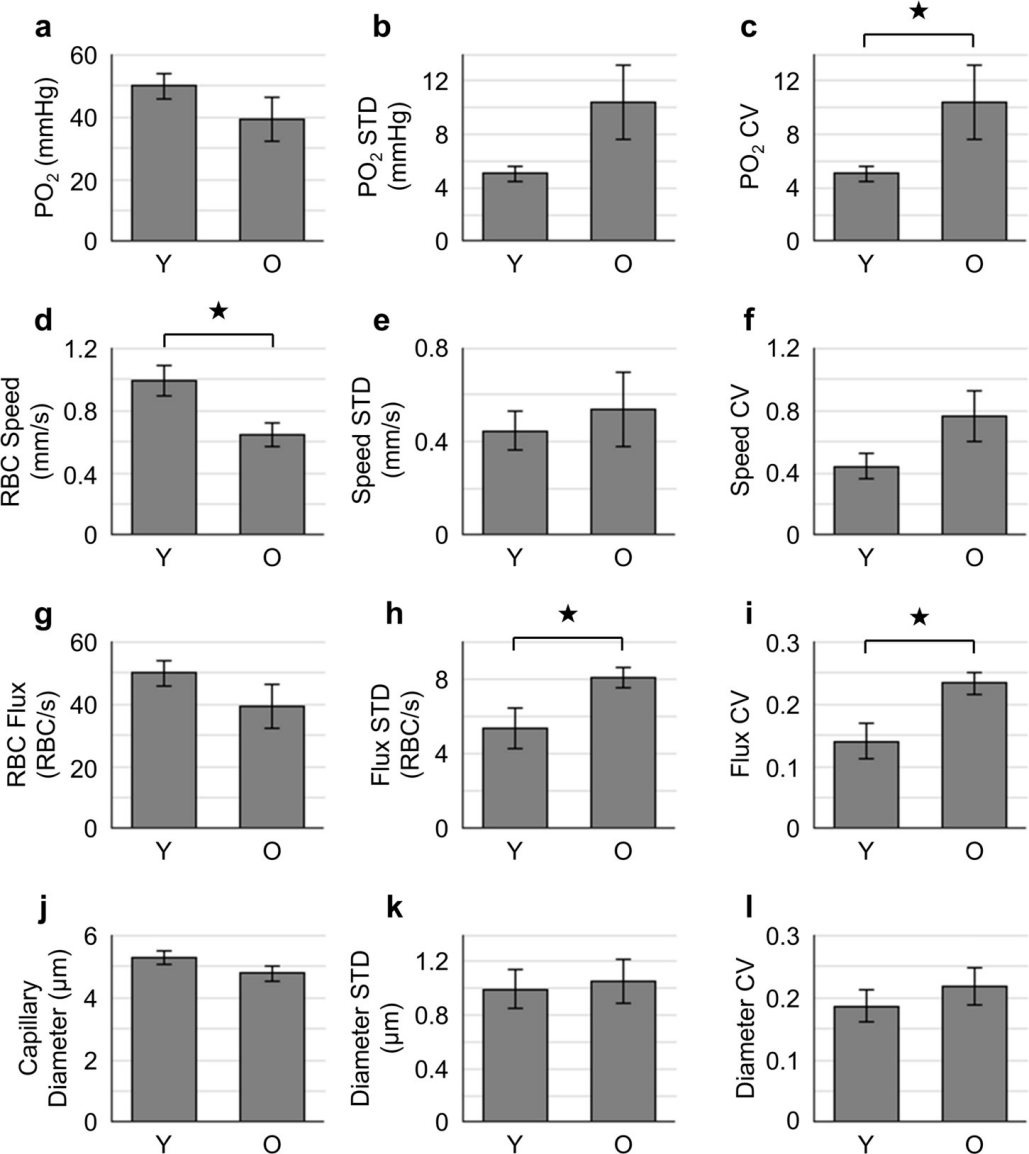
Comparisons of cortical tissue PO_2_, capillary RBC flow and diameter between the young (Y) and old (O) atherosclerotic mice. **a-c.** Absolute tissue PO_2_, PO_2_ STD and CV; **d-f.** absolute RBC speed, speed STD and CV; **g-i.** absolute RBC flux, flux STD and CV; **j-l.** absolute capillary diameter, diameter STD and CV. The data presented in **a-c** was based on 100–200 samples acquired per mouse. The analysis in **d**-**l** was made with the measurements acquired in 15 capillaries per mouse. In **a-l**, for each age group, data were first averaged with all the measurements in each mouse, and then over mice. Data are expressed as mean±SEM. The star symbol indicates significant difference (P<0.05, Student’s t-test).

Data are presented as mean±SEM. The measurements in Figs 2–4 were first averaged over capillaries within each mouse, and then across mice. Statistical comparisons in Fig 4 were made using Student’s t-Test (MATLAB, MathWorks Inc.). P value less than 0.05 was considered statistically significant. Detailed measurement information is indicated in the text and figure legends, where relevant.

## Results

### Cortical capillary RBC flow and diameter positively correlate with oxygenation in adjacent tissue

We employed a home-built two-photon laser-scanning microscope (Fig 1A) to measure the resting PO_2_ in the parenchymal tissue of the somatosensory cortex of urethane-anesthetized young and old atherosclerotic mice. PO_2_ imaging with a 2D grid acquisition geometry (Fig 1C) was performed within a 400 × 400 μm^2^ FOV, typically at two depths per mouse from 150 μm to 250 μm below the brain surface. At each imaging depth, PO_2_ gradients (Fig 1D) were also acquired in tissue adjacent to capillaries to estimate the capillary wall PO_2_. In the same capillaries, whose wall PO_2_’s were estimated, RBC flux, speed, hematocrit and capillary diameter were measured with the perpendicular and longitudinal line-scan techniques (Fig 1E and 1F) [29,37]. See the METHODS section for details about the experiments, data acquisition and analysis.

We first investigated the PO_2_ changes as a function of radial distance from the capillary wall, perpendicular to the capillary axis. The tissue PO_2_ gradients were grouped into three groups having an equal number of capillaries, based on the wall PO_2_ values of the associated capillaries: high (>46 mmHg), median (35–45 mmHg) and low (<35 mmHg). The average PO_2_ gradient in the high wall PO_2_ group exhibited greatest PO_2_ decrease with distance from the capillary wall (ΔPO_2_ = 2.2 mmHg between PO_2_ at 0–5 μm and 15–20 μm from the wall), while such decreasing trend in the median and low wall PO_2_ groups was much smaller (~1 mmHg; Fig 1A).

We next investigated the relations between capillary RBC flow properties, diameter and wall PO_2_. We observed a positive correlation between RBC speed and wall PO_2_ up to speed values of 1.6–2.0 mm/s. The capillary wall PO_2_ reached a plateau at higher speeds (Fig 2B). A similar trend was observed between RBC flux and wall PO_2_ (Fig 2C), but no clear correlation between RBC hematocrit and wall PO_2_ was observed (Fig 2D). Capillary diameter exhibited a strong positive correlation with wall PO_2_ (Fig 2E). Linear regressions were carried out on the data to quantify the correlations. The slopes of the correlations between RBC speed, flux, diameter, and wall PO_2_ (corresponding to the panels **b, c** and **e**) were calculated to be 8.6 mmHg∙s/mm, 0.2 mmHg∙s/RBC and 4.7 mmHg/μm, respectively; and the associated coefficients of determination (R^2^) were 0.08, 0.02 and 0.11, respectively.

Furthermore, RBC flux exhibited strong positive correlation with speed (Fig 3A). A similar trend was observed in RBC flux vs. hematocrit (Fig 3B). RBC hematocrit was found weakly negatively correlated with speed (Fig 3C). In addition, both RBC flux and speed increased with capillary diameter (Fig 3D and 3E). No clear trend was observed between RBC hematocrit and capillary diameter (Fig 3F). Linear regressions were carried out on the data populations to quantify the correlations. The slopes of the correlations corresponding to panels **a-e** are 0.03 mm/RBC, 20.06 RBC/s, -0.21 mm/s, 0.09 mm/s/μm and 0.64 RBC/s/μm; and the R^2^ values are 0.26, 0.08, 0.01, 0.04 and 0.01, respectively.

### Cortical tissue PO_2_ and capillary RBC flow were lower and more heterogeneous in the older atherosclerotic mice

The absolute values, STD and CV of tissue PO_2_, capillary RBC speed, flux, hematocrit and diameter were compared between the young and old atherosclerotic mice. PO_2_ gradients adjacent to capillaries and tissue PO_2_ measurements were also collected using a 2D grid acquisition geometry in the cortical parenchymal tissue adjacent to the capillary bed. The histograms of tissue PO_2_ (combining the PO_2_ gradient and 2D-grid PO_2_ measurements) acquired in the young and old atherosclerotic mice are presented in S1 Fig.

Absolute tissue PO_2_ in the old atherosclerotic mice (39.4±7.0 mmHg) was lower (P = 0.22) than in the young atherosclerotic mice (49.9±4.0 mmHg; Fig 4A). PO_2_ STD and CV in the old atherosclerotic mice were noticeably higher than in the young atherosclerotic mice (Fig 4B and 4C).

Similar changes from old to young were observed in the capillary RBC flow properties. Specifically, absolute RBC speed in the old atherosclerotic mice (0.66±0.08 mm/s) was significantly lower than in the young atherosclerotic mice (0.99±0.10 mm/s; Fig 4D). RBC flux in the old atherosclerotic mice (35.1±1.3 RBC/s) was lower than in the young atherosclerotic mice (39.4±2.1 RBC/s), but did not reach statistical significance (Fig 4G). Both RBC Flux STD and CV were significantly higher in the old atherosclerotic mice than in the young atherosclerotic mice (Fig 4H and 4I). Capillary diameters (Fig 4J) were not significantly different between the two groups despite exhibiting a lower trend in the older mice (Y: 5.3±0.2 μm; O: 4.8±0.2 μm, P = 0.12). No obvious difference was found in RBC hematocrit, and its STD and CV (S2 Fig).

## Discussion

It has been reported that chronic atherosclerosis could cause cerebral hypoperfusion and cerebrovascular disorders [10–12], leading to cognitive impairment [12–15]. In this study we measured PO_2_ in the cortical tissue of a mouse model of atherosclerosis. Mice lacking the LDL receptor develop atherosclerosis spontaneously, after the age of 3 months, without the need of high cholesterol diet [25,26]. The PO_2_ measurements were performed both densely in cortical tissue adjacent to capillaries and over wider areas containing the capillary bed. Capillary RBC speed, flux, hematocrit and diameter were also measured by two-photon imaging of FITC-labelled blood plasma. We analyzed the pairwise relations between tissue PO_2_, and capillary RBC flow properties and diameter. We also performed group comparisons to explore the impact of chronic atherosclerosis on brain oxygenation and capillary RBC flow.

We first characterized the tissue PO_2_ gradient driven by the oxygen diffusion from capillaries (Fig 2A). Tissue PO_2_ gradients around capillaries have been theoretically predicted [40], but not experimental confirmed. We found that around capillaries associated with high wall PO_2_, the gradient was higher when compared to those associated with medium and low wall PO_2_. There is possibility that the capillaries with high wall PO_2_ were topologically and/or physically closer to pre-capillary arterioles in the network, and had higher oxygenation than the capillaries having medium and low wall PO_2_, thus contributing more to brain tissue oxygenation [41]. Next, we focused on the relations between capillary wall PO_2_ and RBC flow and diameter. It has been reported that tissue PO_2_ in proximity to the capillary wall could represent, to some extent, the intra-capillary PO_2_ [42]. Our results in Fig 2 show that higher capillary RBC flux results in a greater oxygenation in the adjacent tissue. This result could be inferred from a previous study, in which a positive relation between capillary RBC flux and intra-capillary PO_2_ was reported [43]. Furthermore, we analyzed the relations between capillary RBC flow parameters and diameter (Fig 3). Our results showed that both RBC speed and hematocrit were positively related with flux, but hematocrit was slightly negatively related with RBC speed, in agreement with the previous studies [37,43]. In addition, RBC speed and flux increased with capillary diameter, but hematocrit had no clear relation to diameter. Similar results have been reported in previous studies in anesthetized rats [37,44] and mice [36,45].

Absolute tissue PO_2_ measured in the capillary bed tended to be lower in the old atherosclerotic mice (Fig 3), although a significant difference was not reached. However, reduced capillary RBC speed, flux and diameter were observed in the old atherosclerotic mice, which differs from previous studies reporting increased RBC speed and flux, and larger capillary diameter in aged mice [29] and rats [44]. Atherosclerosis is associated with vascular endothelial damage [46,47] and cerebral hypoperfusion [11,12] which could decrease capillary diameter thereby limiting RBC flow.

The reduced capillary RBC flow and diameter in the old atherosclerotic mice was accompanied by an increase in the spatial heterogeneity of the same variables (quantified by STD and CV, Fig 4), implying a compromised oxygen delivery to tissue as predicted by the biophysical models [19,20]. Capillary flow pattern disturbances are not unique to this mouse model of atherosclerosis, but were also found to be involved in the progression of neurodegeneration [21]. It was reported that capillary flow heterogeneity could play a crucial role in focal ischemic stroke, as it could induce local hypoxia and neuronal death, without a detectable decrease in the absolute global cerebral blood flow [48].

The origin of capillary RBC flow heterogeneity remains difficult to identify. We have found smaller average capillary diameter but larger diameter heterogeneity in the old atherosclerotic mice. As capillary RBC flow was reported to be correlated positively with diameter and intra-capillary PO_2_ [43,44], our findings about diameters are in good agreement with the findings about capillary RBC flow and tissue PO_2_ (Figs 2 and 3). Therefore, these results imply that a disturbed flow pattern might essentially originate from microvascular structural changes [49].

Limitations exist in this study. Although the animal physiology was carefully controlled in experiments, anesthesia inevitably introduce hemodynamic changes relative to the awake state [50], thus adding variability to the measurements. Furthermore, we cannot exclude the possibility of an anesthetic effect with age which remains a limitation of this study. Nonetheless, in our previous studies in either anesthetized [44] or awake wild-type animals [29], comparisons of the absolute capillary blood flow and flow heterogeneity between the young and old mice followed similar trends, implying that anesthesia may not differentially affect the brain physiology among different age groups. Measurements in age-matched wild-type mice were not available in this study. In the previous work with the same atherosclerotic model, difference in cerebral blood flow between the 3-month and 6–12 month mice was found significantly different [28], but the aging effect on cerebral blood flow, oxygenation and capillary blood flow properties in the healthy wild-type mice occurred much later (e.g., 2-year old) [29].

To conclude, our study for the first time demonstrated that tissue PO_2_ was positively correlated with capillary RBC flow and capillary diameter. In addition, we found lower tissue PO_2_, lower capillary RBC flow and smaller capillary diameter in the old atherosclerotic mice when compared to younger mice. We also found more spatially heterogeneous distributions of tissue PO_2_, RBC flow properties and capillary diameter in the old atherosclerotic mice when compared to the younger mice. Overall, our results confirm a detrimental impact of atherosclerosis on brain oxygenation and microvascular function.

## Supporting information

S1 Fig Histograms of tissue PO_2_ measured in the young (Y) and old (O) atherosclerotic mice. The tissue PO_2_ distribution in the old atherosclerotic mice is broader and shifted towards the lower PO_2_ values when compared to the young mice.
(TIF)

S2 Fig Comparison of capillary RBC hematocrit (Hct) between the young (Y) and old (O) atherosclerotic mice. **a-c.** Comparisons of absolute Hct, Hct STD and CV, respectively. The analysis was made with the measurements acquired in 15 capillaries per mouse. The data were first averaged with all the measurements in each mouse, and then over mice. Data are expressed as mean±SEM. No significant difference was found (Student’s t-test).
(TIF)
