# Supplementary figures and images for: Atherosclerosis is associated with a decrease in cerebral microvascular blood flow and tissue oxygenation
Source: PLoS One. 2019 Aug 30;14(8):e0221547. doi: 10.1371/journal.pone.0221547 (PMC6716780; doi:10.1371/journal.pone.0221547)

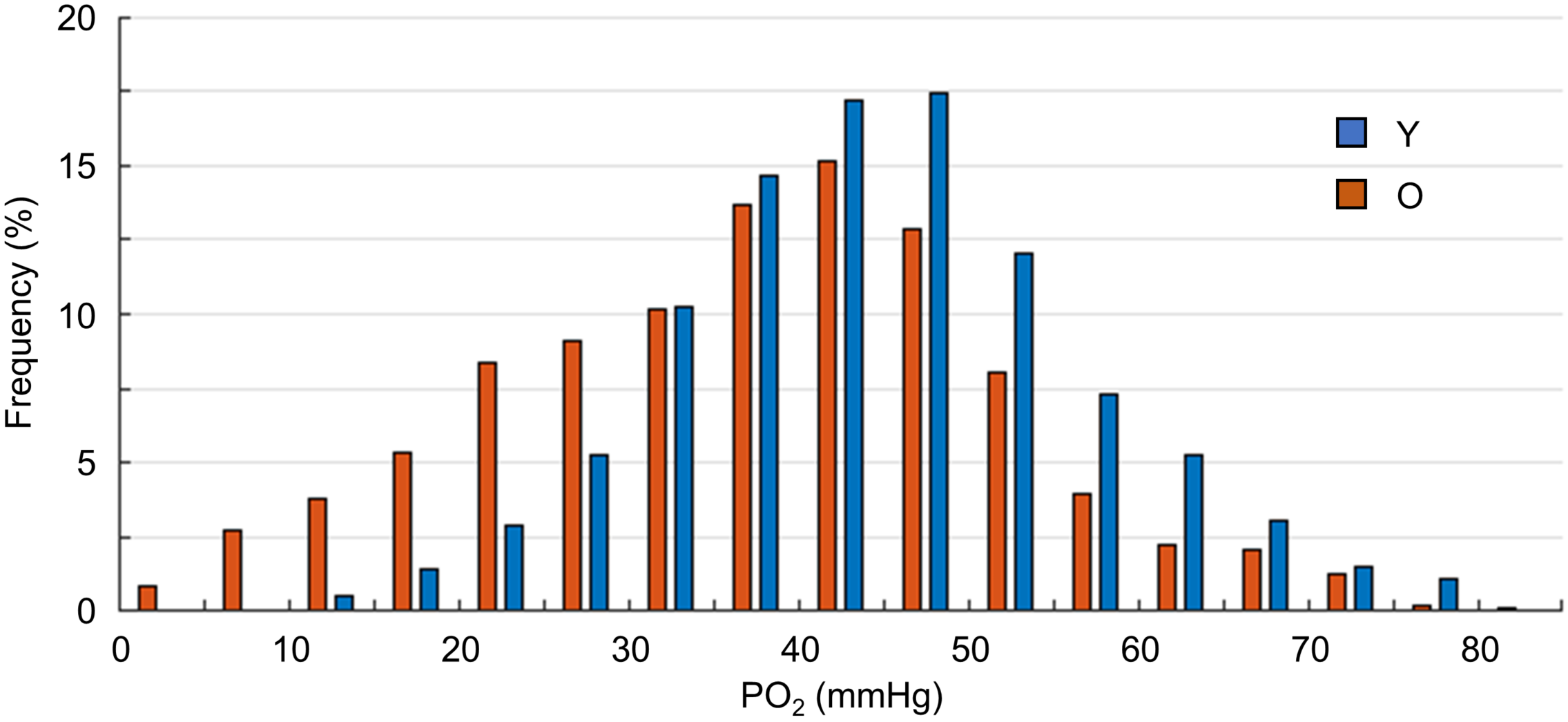

Supplement: S1 Fig — The tissue PO2 distribution in the old atherosclerotic mice is broader and shifted towards the lower PO2 values when compared to the young mice. (TIF) [file pone.0221547.s001.tif]

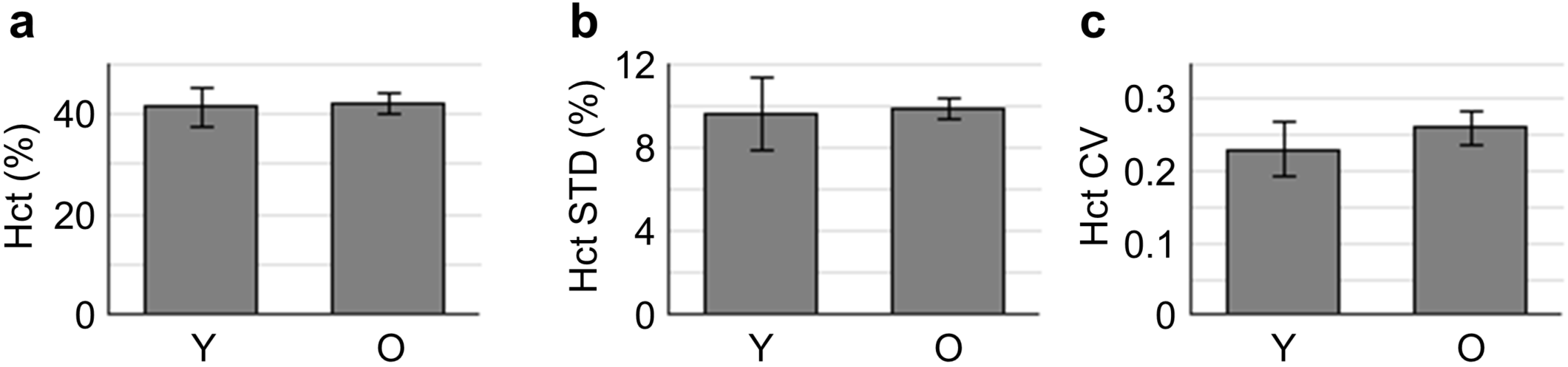

Supplement: S2 Fig — a-c. Comparisons of absolute Hct, Hct STD and CV, respectively. The analysis was made with the measurements acquired in 15 capillaries per mouse. The data were first averaged with all the measurements in each mouse, and then over mice. Data are expressed as mean±SEM. No significant difference was found (Student’s t-test). (TIF) [file pone.0221547.s002.tif]
